# The change of gait on shoes sole form

**DOI:** 10.1186/1757-1146-7-S1-A100

**Published:** 2014-04-08

**Authors:** Se won Yoon, Jeong woo Lee, Soo ji Park, Woong sik Park, Woon su Cho

**Affiliations:** 1 Department of physical therapy, Kwangju women’s university, Kwangju, 506-713, Korea; 2 Department of physical therapy, Graduate school, Kwangju women’s university, Kwangju, 506-713, Korea; 3 Department of occupational therapy, Kwangju women’s university, Kwangju, 506-713, Korea; 4 Department of physical therapy, Nambu university, Kwangju, Korea

## Background

Preceding studies reported that differences in pressure distribution according to shoe type [1] and there were many comparative analyses of motor mechanics between travel shoes and general running shoes [2], but they were confined to research on pressure distribution of foot and studies on muscle activity and gait cycle depending on shoes heel were rare.

Therefore, the purpose of this study was to examine change of gait (gait parameter) on shoes sloe form through gait analyzer.

## Method

This study selected 12 normal female in their twenties. Gait analyzer is composed of two sending and receiving bars of 5cm long and webcam and the width of both bars was 1m. The subject’s gait was sensed between sending and receiving bars and information of temporal and spatial variables was collected. Webcam was used to save image information and synchronize the subject’s gait exactly.

All subjects of this study put three kinds of shoes including high heel, MBT shoes and house shoes and were measured once respectively. Experiment was made of the following procedures. The researcher demonstrated 5m gait personally before subjects gait. And then the researcher said to subjects “walk please”, subjects were put on three kinds of shoes once and walk 5m on gait analyzer. Subjects were measured by putting a pair of shoes and allowed to take a rest for 2 min.

## Result

As result of change of gait parameter of left and ring lower extremity on shoes sole form, step, single support, load response were showed significantly difference (p<0.05). But stance phase, swing phase, gait time were showed no significantly difference. As result of change of gait parameter of double support phase on shoes sole form, stride, double support were showed significantly difference (p<0.05). But gait cycle, gait rate were showed no significantly difference.

## Conclusion

Step and stride of gait parameter showed shorten when wearing high heel. We think that because our subjects were normal female in their twenties adaptive high heel height, step and stride of gait parameter were shorten. MBT shoes showed the highest load response of gait parameter in three type shoes, because MBT shoes activate tibialis anterior and gastrocnemius. Therefore we suggest that lower limb diseases patients consider gait parameter when helpful shoes select.
